# Inflammasome: a new trigger of Alzheimer's disease

**DOI:** 10.3389/fnagi.2014.00080

**Published:** 2014-05-06

**Authors:** Tara Saco, Prasanna T. Parthasarathy, Young Cho, Richard F. Lockey, Narasaiah Kolliputi

**Affiliations:** Division of Allergy and Immunology, Department of Internal Medicine, Morsani College of Medicine, University of South FloridaTampa, FL, USA

**Keywords:** inflammasomes, Alzheimer Disease, inflammation, injury severity score, IL-1beta, caspases

Alzheimer's disease is an insidious and dementing illness currently affecting approximately 5 million Americans over the age of 65, and today, someone in the US develops Alzheimer's disease every 68 s which is expected to drop to 33 s in the year 2050 (Thies and Bleiler, 2013). Although the scientific community has learned much about the pathogenesis of Alzheimer's disease in recent years, treatments to prevent progression and reverse the effects of this disease have yet to be developed. Current therapies are primarily indicated for symptomatic control, but they do not have the capacity to inhibit or lead to regression of the pathologic changes contributing to Alzheimer's disease. Therefore, current research into this condition is primarily focused on developing targeted therapies against genes involved in the development of Alzheimer's disease. Currently, an avenue that is being focused on and should be given further attention is the epigenetic mechanisms contributing to the pathophysiology of Alzheimer's disease.

The main contributing factor in the development of Alzheimer's disease is the deposition of β-amyloid in the brain, especially in the hippocampus (Heneka et al., 2013). Previous research which focused on chronic medical conditions involving β-amyloid pathology has shown that inflammatory mechanisms may be stimulated by β-amyloid deposition (Halle et al., 2008). Formation of β-amyloid plaques stimulates the production of inactive IL-1β, an inflammatory cytokine (Prinz et al., 2011). However, the mechanism that initiates inactive IL-1β processing in Alzheimer's disease is not clearly defined. It was recently reported that caspase-1-mediated processing of IL-1β is mediated by inflammasomes in various pathological conditions (Kolliputi et al., 2010, 2012; Fukumoto et al., 2013). Inflammasomes, such as the NLRP3 inflammasome, detect the inflammatory aggregates of β-amyloid and inactive IL-1β, and respond by secreting caspase-1 (Casp-1) to activate IL-1β (Heneka et al., 2013; Qazi et al., 2013). This leads to the creation of an inflammatory environment around the plaque, which downregulates amyloid precursor protein (APP) degradation, as well as decreased destruction of β-amyloid plaques by microglia (Figure 1). Although it can be surmised that this mechanism may contribute to the development of Alzheimer's disease, its involvement has not been experimentally demonstrated (Heneka et al., 2013). NLRP3 inflammasome activation by β-amyloid in microglia is necessary for maturation of IL-1β and subsequent inflammatory events; however, the role of NLRP3 activation in Alzheimer's disease *in vivo* is still unknown (Heneka et al., 2013). A recent study by Heneka et al. suggests that the NLRP3 inflammasome has a role in Alzheimer's disease by demonstrating increased caspase-1 expression levels in brains with Alzheimer's disease.

**Figure 1:**
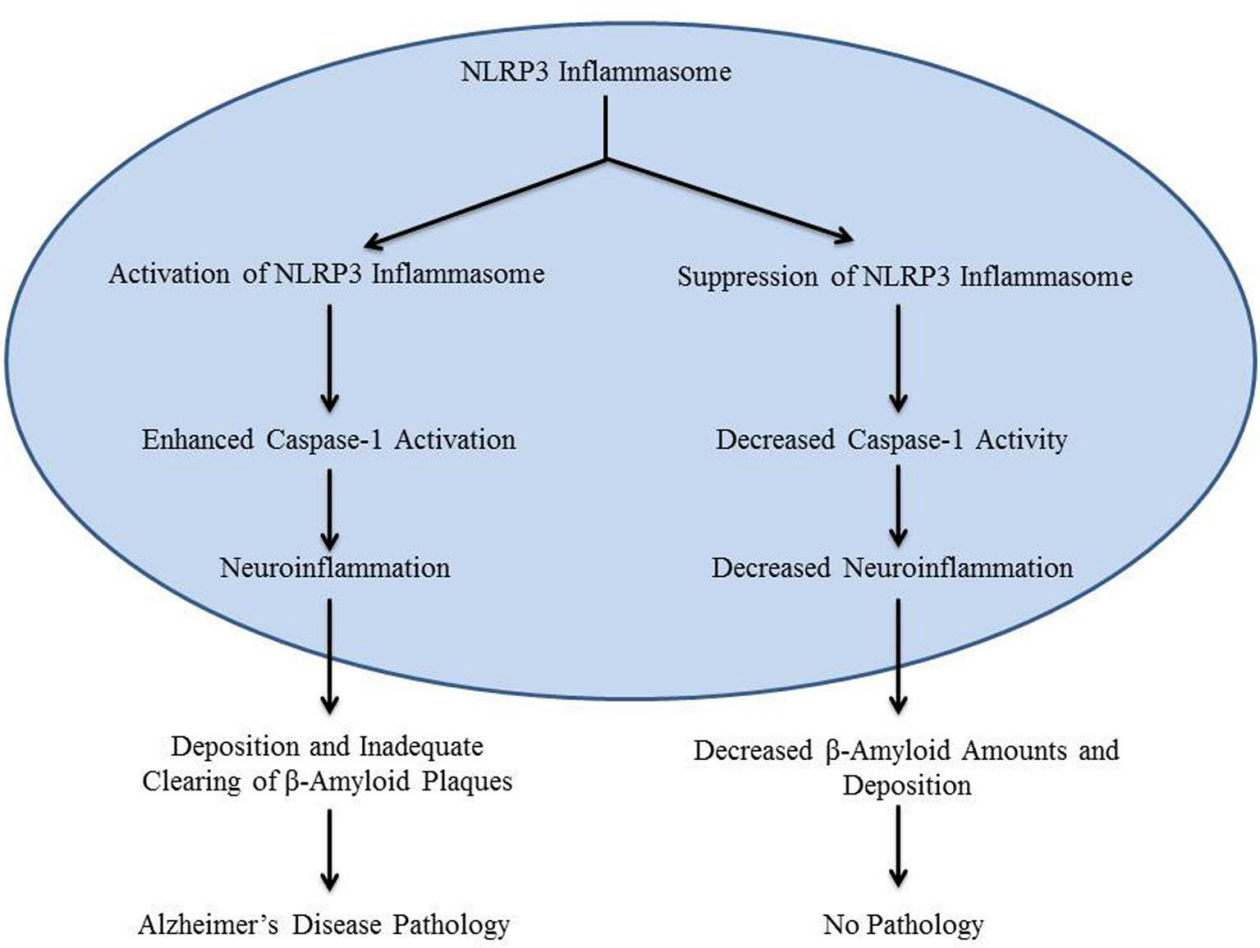
**The diagram is a schematic representation of the role of NLRP3 inflammasome in Alzheimer's disease**.

Recently, Heneka et al. conducted a study to determine whether or not NLRP3 inflammasome activation contributes to the pathogenesis of Alzheimer's disease. APP/Presenilin-1 (PS1) mice, which develop symptoms similar to Alzheimer's disease, were crossed with NLRP3^−/−^ or Casp-1^−/−^ mice. The quantity of cleaved Casp-1 in the brains of the offspring was then measured in comparison to the APP/PS1 mice. The researchers also examined the differences in the level of spatial memory impairment between these groups and found that in APP/PS1/NLRP3^−/−^ and APP/PS1/Casp-1^−/−^ mice, the level of cleaved Casp-1 was markedly decreased when compared to APP/PS1 mice, while IL-1β levels were consistent between the groups (Heneka et al., 2013). This diminished Casp-1 level correlated with preserved spatial memory function in the APP/PS1/NLRP3^−/−^ and APP/PS1/Casp-1^−/−^ mice, as opposed to the significant spatial memory impairment seen in the APP/PS1 mice (Heneka et al., 2013). The researchers also compared the level of long-term potentiation (LTP), which is analogous to synaptic plasticity, in the hippocampi of these mice. The APP/PS1 mice were found to have decreased hippocampal synaptic plasticity, while the hippocampal synaptic plasticity in the APP/PS1/NLRP3^−/−^ and APP/PS1/Casp-1^−/−^ mice was preserved (Heneka et al., 2013).

The role of the NLRP3 inflammasome in behavioral changes and cognitive deficiencies associated with Alzheimer's disease was also examined in the study by Heneka et al. APP/PS1 underwent behavioral analysis and were found to have delayed habituation and increased psychomotor agitation. Conversely, APP/PS1/NLRP3^−/−^ mice exhibited decreased locomotion and behavioral changes, further supporting the fact that the NLRP3 inflammasome places a large part in the development of cognitive and behavioral symptoms seen in Alzheimer's patients.

The study by Heneka et al. also assessed the effect of the NLRP3 inflammasome on the phagocytic activity of microglia. Studies have shown that large numbers of microglia surround β-amyloid plaques in Alzheimer's patients. However, it is also well known that inflammatory cytokines inhibit microglial phagocytosis. Thus, Heneka et al. suggested that the NLRP3 inflammasome contributes to ineffective microglial phagocytosis by leading to increased expression of inflammatory cytokines. After isolating microglia from the APP/PS1 mice, APP/PS1/NLRP3^−/−^ mice, and APP/PS1/Casp-1^−/−^ mice, Heneka et al. found that microglial phagocytosis of β-amyloid was more efficient as it doubled in APP/PS1/NLRP3^−/−^ mice and APP/PS1/Casp-1^−/−^ mice when compared to APP/PS1. The deficiency in the NLRP3 inflammasome or Casp-1 did not change the overall amounts of APP but rather reduced the levels of β-amyloid aggregates. These results provide additional evidence that the NLRP3 inflammasome leads to pathologic deposition and ineffective clearing of β-amyloid in Alzheimer's patients.

Based on these results, the authors conclude that introducing therapeutic treatments targeting the NLRP3 inflammasome may have a beneficial effect on patients with Alzheimer's disease as NLRP3 activation may contribute to the pathogenesis of Alzheimer's disease in humans. The authors also suggest that activation of NLRP3 enhances Alzheimer's disease and may be involved in synaptic dysfunction, cognitive impairment, and the restriction of microglial clearance functions (Heneka et al., 2013). Therefore, human trials should be conducted to determine the validity of this hypothesis. The experiment by Heneka et al. provides an excellent model for the development of possible future therapies for Alzheimer's patients. Animal and human studies examining the safety and effectiveness of targeted NLRP3 or Casp-1 inhibitors should be further pursued as the results reveal an important role of the inflammatory activators NLRP3 inflammasome/caspase-1 in Alzheimer's disease pathogenesis and could represent improved therapeutic treatments for millions of Alzheimer's disease patients.

## Conflict of interest statement

The authors declare that the research was conducted in the absence of any commercial or financial relationships that could be construed as a potential conflict of interest.
